# Integration Site and Clonal Expansion in Human Chronic Retroviral Infection and Gene Therapy

**DOI:** 10.3390/v6114140

**Published:** 2014-10-31

**Authors:** Heather A. Niederer, Charles R. M. Bangham

**Affiliations:** Department of Immunology, Wright-Fleming Institute, Imperial College London, London W2 1PG, UK; E-Mail: h.niederer@imperial.ac.uk

**Keywords:** HTLV-1, gene therapy, gammaretroviral vector, integration site

## Abstract

Retroviral vectors have been successfully used therapeutically to restore expression of genes in a range of single-gene diseases, including several primary immunodeficiency disorders. Although clinical trials have shown remarkable results, there have also been a number of severe adverse events involving malignant outgrowth of a transformed clonal population. This clonal expansion is influenced by the integration site profile of the viral integrase, the transgene expressed, and the effect of the viral promoters on the neighbouring host genome. Infection with the pathogenic human retrovirus HTLV-1 also causes clonal expansion of cells containing an integrated HTLV-1 provirus. Although the majority of HTLV-1-infected people remain asymptomatic, up to 5% develop an aggressive T cell malignancy. In this review we discuss recent findings on the role of the genomic integration site in determining the clonality and the potential for malignant transformation of cells carrying integrated HTLV-1 or gene therapy vectors, and how these results have contributed to the understanding of HTLV-1 pathogenesis and to improvements in gene therapy vector safety.

## 1. Introduction: Why Is the Retroviral Integration Site Important?

In both the germline (endogenous retroviruses) and in somatic cells (exogenous viruses; gene therapy vectors), retroviruses can integrate and persist in humans for years. Certain retroviruses are major pathogens, while others have played important roles in genome evolution [1,2]. The location of retroviral integration within the genome was originally thought to be random, but a large body of evidence now shows that integration is non-random and that the integration site can influence the expression of the integrated provirus. Each retrovirus has its own particular characteristics of integration site preference and interaction with the host genome. The human deltaretrovirus Human T-cell Lymphotropic Virus type 1 (HTLV-1) is maintained in T lymphocytes by continued replication of infected cells for the lifetime of the infected individual. Retroviral and lentiviral vector integration sites are likewise maintained by replication of transduced cells for months or years in human recipients. Persistent proliferation can lead to selective expansion or survival of certain transduced or infected cell clones, and certain clones may undergo uncontrolled proliferation, leading to malignant disease. Adult T-cell leukaemia/lymphoma (ATL) develops in up to 5% of HTLV-1 infected patients, and 25% of patients treated in early gammaretroviral trials for SCID-X1 developed leukaemia [3,4]. In this review we consider the interactions between the host genome and both HTLV-1 and retroviral gene therapy vectors, and consider how work from each field can inform the other.

## 2. HTLV-1

HTLV-1 and the closely related HTLV-2 both preferentially infect T cells *in vivo*. Transmission between individuals occurs through infected breast milk, blood or semen [5]. Efficient viral transmission requires cell-to-cell contact, which triggers polarisation of the microtubule organising centre and transfer of HTLV at the virological synapse [6]. Membrane fusion and viral entry involves interaction of HTLV envelope proteins with cellular receptors including GLUT-1 [7].

Following cell entry, the HTLV-1 viral RNA genome undergoes reverse transcription, and a single copy [8] of the resulting DNA provirus integrates into the host genome. Expression of viral genes including Tax and HBZ can drive proliferation of HTLV-1-infected cells (reviewed in [9,10]), forming expanded clonal populations, each clone defined by a single genomic integration site. In infected individuals, integrated HTLV-1 provirus is predominantly found in CD4^+^ T cells [11], while HTLV-2 favours CD8^+^ cells [12]. There is evidence *in vitro* that this preference may be due to differential proliferation of infected cells rather than a differential tropism [13].

Although expression of HTLV-1 genes provides a proliferative advantage to the infected T-cell clone, it also leads to viral epitope display on host HLA class I molecules, making the infected cell a target of the host cytotoxic lymphocyte (CTL) response. In chronic infection, viral gene products from the HTLV-1 positive strand (including Tax) are rarely detected in PBMCs directly *ex vivo* [14,15], suggesting that it is advantageous for HTLV-1 to evade the strong immune response to Tax by silencing expression of the positive strand. However, the high frequency of activated CTLs that recognize Tax antigens [16,17] implies that Tax protein is abundantly expressed *in vivo*, at least at some times and in some cells. How Tax silencing and re-expression are regulated is not understood.

In contrast to Tax, expression of HBZ from the minus strand is maintained at a low level *in vivo* and is detected in PBMCs directly *ex vivo* [18]. HBZ appears to favour proliferation of the infected cell [18]. Although DNA methylation of the 5’ LTR frequently suppresses expression of Tax, the 3’ coding part of the HTLV-1 genome and the 3’LTR remain unmethylated [19]. HBZ is poorly presented on most HLA class I molecules, and only ~30% of individuals have a detectable CTL response to HBZ [20]. Thus the deleterious consequences for the virus of HBZ expression may be less than Tax expression, while maintaining cell proliferation. Those individuals who do have HLA class I molecules able to present HBZ have a significantly lower PVL and a reduced risk of the HTLV-1-associated inflammatory disease HAM/TSP [21]. The survival and abundance of an infected clone depend on its pattern of expression of viral genes and the balance between the positive (proliferative) and negative (immune selection) effects of that expression.

## 3. Gammaretroviral and Lentiviral Gene Therapy Vectors

The retroviral DNA integrated in the host genome is transmitted to daughter cells at mitosis. This makes retrovirus-based gene delivery systems an attractive method to stably introduce new genetic information into host cells, for instance to correct gene defects. Substantial progress has been made in developing retrovirus-based gene therapy vectors, especially for primary immunodeficiency disorders. In cases where HLA-matched donor bone marrow is not available, patient-derived autologous hematopoietic stem cells (HSC) can be isolated, transduced and transplanted back into the patient [22]. Diseases that are suitable targets for this approach include two forms of severe combined immunodeficiency (SCID), where the disease is due to a single defective gene, of moderate size, which can be expressed in leukocytes. In addition, the transgene may give a selective advantage to the transduced cells *in vivo*, which increases the success of therapy [23].

Gene therapy vectors based on the gammaretrovirus murine leukaemia virus (MLV) have been used in a number of gene therapy trials following the first trials in SCID-X1 [24,25]. The retroviral vector system (reviewed in [26]) comprises a vector with the transgene of interest flanked by the long terminal repeats (LTR) and the packaging signal, and two helper plasmids encoding the viral Gag/Pol and Env proteins, respectively. Target cell tropism can be altered by using a different viral Env, in a process known as pseudotyping. Since the helper plasmids do not contain the retroviral packaging sequence, the final packaged virus does not contain RNA encoding these elements, which precludes recombination events. Although transgene expression can be driven by the MLV 5’LTR enhancer/promoters, LTRs from the Spleen-Focus Forming Virus (SFFV) and Myeloproliferative Sarcoma Virus (MPSV) have been used to provide stronger expression in hematopoietic cells.

Recently, HIV-based self-inactivating vectors have been used in a number of trials [27,28,29,30]. HIV-based vectors (reviewed in [31]) are advantageous in that they can transfect resting cells, partly owing to an efficient mechanism of nuclear import [32]. Advanced lentiviral vectors incorporate several improvements, including a self-inactivating LTR with a significant deletion in the U3 (including the TATA box) in the 3’LTR of the retroviral plasmid. Upon integration, the deletion version of U3 is copied into both LTRs, thus limiting effects of the viral LTR promoter on the genome flanking the integrated vector. The transgene is typically driven from an internal promoter incorporated in the vector. Lentiviruses require the accessory proteins Tat and Rev, but in third generation vectors the requirement for Tat is removed, and Rev is expressed on a fourth plasmid to further minimise recombination [33].

## 4. High-Throughput Mapping of Retroviral Integration Sites

Ligation-mediated PCR (LM-PCR) and linear amplification-mediated PCR (LAM-PCR) have been the most frequently used systems to determine retroviral provirus or vector integration site. Both techniques involve ligation of a linker DNA cassette to fragmented genomic DNA, which enables PCR amplification between known sequences in the viral LTR and the linker. High-throughput sequencing (for instance Illumina technology) is then used to sequence the host integration site DNA between the LTR and the flanking restriction site [34,35]. LAM-PCR is sensitive, and is useful in situations with limited quantities of DNA. Both techniques initially relied on restriction enzymes to digest the genomic DNA. However, the use of restriction enzymes confers a bias: the integration sites may fail to amplify if the nearest restriction site lies too far from the integration site, because short DNA fragments are preferentially amplified. This bias can lead to an imbalance in the integration site profile, which is only partly alleviated by the use of several restriction enzymes [36]. The accuracy of quantifying clone abundance from the number of sequenced reads alone is debated. Estimates of relative clone abundance by read counts in restriction enzyme-based techniques have been shown to differ from qPCR estimates, probably because of PCR bias influenced by amplicon length and GC content [37]. However, the correlation between clonal abundance and the sequence read count also depends on sequencing depth, the quantity of DNA input, and the strategy used for genome-wide retrieval.

Recent modifications to both LM-PCR and LAM-PCR have addressed these issues. A modified protocol for nrLAM-PCR has eliminated the need for restriction enzymes by ligating single stranded linker oligonucleotides to linear single-stranded amplified vector-genome junctions [38]. nrLAM-PCR is less sensitive than LAM-PCR, but comparable to LM-PCR [38]. In a particularly powerful advance to LM-PCR, ultrasonic shearing of the genomic DNA [39] is used instead of restriction enzyme digestion. Since the resulting shear sites are virtually random, this method has two major benefits. First, it abolishes the preferential amplification of integration sites that lie near a given restriction site. Second, each “sister” cell of the same infected clone (*i.e.*, sharing the genomic integration site) has a shear site in its genome at a unique distance from the viral integration site. This makes it possible to count the sequenced sister cells of each clone. A small correction is needed for very abundant clones, because identical shear sites can occur by chance [39,40]. Further refinements include using random barcodes to improve the accuracy of quantification of highly abundant clones [41]. Finally, phage Mu can be used to introduce the adaptor sequence in a near-random way, also eliminating the need for restriction enzymes [42].

## 5. Retroviral Integration Site Preferences

### 5.1. Primary DNA Target Sequence

**Figure 1:**
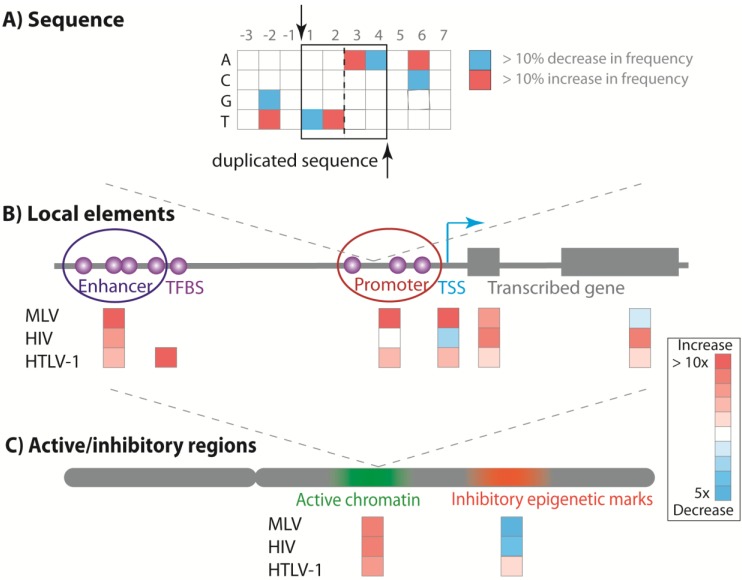
Genomic features associated with retroviral integration sites. The site of retroviral integration is determined by factors at several physical scales. (**A**) Each viral integrase has a weak preference for a short characteristic genomic sequence (the MLV preference is illustrated here, based on data in [44]); (**B**) Host proteins interact with the viral integrase and guide integration into genes and regulatory elements, such as promoters and enhancers, and near to (100–1000 bp) transcription start sites (TSS) and transcription factor binding sites (TFBS); (**C**) The frequency of integration differs between regions of the genome marked respectively by inhibitory and activatory epigenetic marks. The heat map marks a typical fold change in integration frequency compared with random sites, based on data in [39,49,55].

Retroviral integration is not random at the primary sequence level. MLV and derived gene therapy vectors share weakly conserved, apparently palindromic target sequences with symmetry around a 4 bp target sequence that is duplicated during integration [43,44]. This targeting is determined by the viral integrase alone [45]. The target sequence of HIV is centred on the five nucleotides that are duplicated during integration [43,44], whereas the HTLV-1 and HTLV-2 target sequences are symmetrical around six duplicated nucleotides [46,47,48] (Figure 1A).

### 5.2. Local Genomic Features

The conserved DNA sequence does not completely describe the observed retroviral integration site profile: for example, MLV integration sites are strongly clustered in the genome. Recent high-throughput sequencing of MLV integration sites (>1 million sites sequenced per study) demonstrated that at least half of the sequenced MLV integration sites could be assigned to tight clusters that covered less than 2% of the genome [49,50].

MLV preferentially integrates near transcriptional start sites (TSS) and gene regulatory elements such as promoters and enhancers [49,50,51,52]. A recent study of an MLV-based vector demonstrated that 87% of observed integration sites (N > one million) were clustered near peaks of the epigenetic mark H3K4me1, which is characteristic of enhancers, and ~15% lay within 1Kb of an active TSS [49]. A sharp decrease in integration frequency immediately proximal (−80 to +20 bp) to a TSS was attributed to nucleosome-free DNA (and the lack acetylated histone tails) at the TSS [49] or the presence of a host transcription complex [52]. MLV had only a slight bias towards integration within a gene, primarily within the first 10% of the gene length [52]. In contrast, HIV-1 integration is strongly biased towards transcriptional units and avoids TSS [53,54]. Most (75%–80%) HIV-1 integration sites were in transcriptional units [54]. HIV integration sites are evenly distributed within a gene body, except for the first 10% of the gene length closest to the TSS, which is disfavoured [52] (Figure 1B).

Like MLV, HTLV-1 integration near TSS is also favoured. This bias is greatest at approximately 1Kb from the TSS, and diminishes sharply within 100 bp from the TSS [55], perhaps because host cell factors bound near the TSS block access to the host DNA by the viral pre-integration complex. HTLV-1 integration is also mildly favoured within genes [39,47]. The strongest observed bias in HTLV-1 integration is for within 100–1000 bp of certain transcription factor binding sites (TFBS): the odds ratios *versus* random integration can be extremely high (>100 in each case for STAT1, p53 and HDAC6) [55], although each TFBS only accounts for a small proportion of the total number of integration sites. Although certain host genomic features are moderately favoured, in analysing over 250,000 integration sites of HTLV-1 in both *in vitro* and *in vivo* infection [39,55,56,57,58], we have found no evidence of recurrent (common) proviral integration sites or strong clusters (“hot spots”) of integration sites, in contrast to the hot spots reported in HIV-1 infection [53].

### 5.3. Transcriptionally Active Chromatin

HTLV-1 preferentially integrates in areas containing active epigenetic marks (~twofold higher than random frequency of integration). *In vitro*, we observed preferential HTLV-1 integration in a human T cell line (Jurkat) within 10 Kb of epigenetic marks associated with active promoters or enhancers [39,47]. There was also a weak preference for areas enriched locally in inhibitory epigenetic marks associated with heterochomatin [39] (Figure 1C).

When combinations of histone marks were used to define certain human genome “states” [59], strong active enhancers (marked by H3K4me1, H3K4me2, H3K4me3, H3K27ac and H3K9ac) were enriched >40-fold in MLV integrations over random, compared to a 25-fold enrichment at active promoters and a fivefold enrichment within 5 kb of a TSS [50]. HIV-1 integration is also strongly associated with transcriptionally active regions, and the frequency of integration within a gene increases with transcription of the gene [53,54]. Lentiviral vectors also show altered integration site preferences depending on the activity status of the transduced cells [60,61].

### 5.4. Host Factor Interaction with the Viral Integrase

The viral integrase is the major determinant of the viral integration site preferences: a chimeric HIV-based virus containing the integrase of MLV mimicked the MLV integration preference [62]. The distribution of HIV-1 integration sites is influenced by the interaction between the HIV integrase and host proteins, the best characterised of which is lens epithelium-derived growth factor (LEDGF) (reviewed in [63]). The N-terminal PWWP domain of LEDGF interacts with chromatin marked with H3K36me3 [64,65], which is associated with actively transcribed gene bodies [66]. The HIV preintegration complex reaches the nucleus via the nuclear pore complex in non-dividing cells, and does not require the dissolution of the nuclear membrane during mitosis [32]. The integration site distribution of HIV is influenced by nuclear pore proteins including RanBP2/Nup358 and the karyopherin Transportin-3 [67], which may also result in delivery of the preintegration complex to chromatin at particular locations in the nucleus. LEDGF fusion proteins, where the N-terminal domain is replaced with chromatin binding domains from other proteins, can re-direct integration [68]. Paired with a modified lentiviral integrase, this may facilitate altered targeting *in vivo* [69].

The strong bias in MLV integration has been explained in part by the recent work of three groups that demonstrated the interaction of the MLV integrase with the bromodomain and extraterminal (BET) proteins (BRD2, BRD3, BRD4) [70,71,72]. BET proteins are epigenetic “readers” which bind highly acetylated H3 and H4 histone tails via two N-terminal bromodomains [73,74]. In addition to two bromodomains, BET proteins have an extraterminal (ET) protein-protein interaction motif, which interacts with the C-terminal domain of the MLV integrase [71]. A fusion between the BET integrase binding domain and LEDGF chromatin binding domain was sufficient to retarget integration of MLV towards the HIV integration profile [70].

As has been demonstrated for other retroviruses, it is likely that the HTLV-1 integrase is guided to preferred locations in the genome by interactions between the integrase and host proteins. Identifying the putative partners of the HTLV-1 integrase is a subject of current research.

## 6. Integration Site Clonal Expansion *in Vivo*

### 6.1. Disease-Associated Clonal Expansion Is Observed in Vivo

Following initial integration, infected or transduced cells persist for years *in vivo* through mitotic division to become clonal populations. Cells containing certain integration sites can undergo selective expansion *in vivo*, resulting in abundant clonal populations [39,75].

We have sequenced HTLV-1 integration sites from PBMCs in individuals with chronic HTLV-1 infection, both asymptomatic individuals and those with the inflammatory disease HAM/TSP or the HTLV-1 associated malignancy ATLL, and in people co-infected with HTLV-1 and *Strongyloides stercoralis* [39,55,56,57,58]. Using our sonication LM-PCR approach, we quantified the relative abundance of HTLV-1-infected T cell clones and, from the subject’s proviral load, the absolute abundance of each clone. Integration site analysis of individual infected patients can detect thousands of integration sites from an input of 10 µg of genomic DNA. We devised a mathematical technique, based on repeated subsampling, to estimate the number of integration sites in the circulation of infected patients [76].

There are of the order of 10,000 integration sites in a typical HTLV-1 infected person [58]. This implies that each individual has viral DNA in thousands of different genomic contexts, both in the linear genome and in intra-chromosomal and inter-chromosomal interactions, and in proximity to features such as the nuclear envelope or nucleolus. Clones detected by LM-PCR in asymptomatic individuals range in absolute abundance from <0.1 to ~100 cells per 10,000 PBMCs; less abundant clones are undetectable without an increase in the quantity of genomic DNA sampled and in the sequencing depth [39]. Infected CD8^+^ clones appear to make up a disproportionately high number of the most highly expanded clones [48]. The distribution of the clone abundances can be quantified by an oligoclonality index (OCI; based on the Gini index for inequality): infected cell populations dominated by a single clone approach an OCI of one, and populations with similarly sized clones approach an OCI of 0 [39]. The inflammatory disease HAM/TSP is associated with a higher number of distinct clones in the circulation than in asymptomatic carriers, but the OCI does not differ between the two groups [39]. The greater number of infected T-cell clones in patients with HAM/TSP is believed to result from a less effective host CTL response [77]. Highly expanded clones were observed in individuals co-infected with *Strongyloides*, but the total number of clones in the blood was not significantly different from asymptomatic individuals [57]. In ATL, a single presumed malignant clone is overwhelmingly dominant in 80% of cases, with an underlying polyclonal population [56]. In nearly ¼ of ATL cases in a recent study, there was one or more subdominant “intermediate” sized expanded clone, with an absolute abundance greater than observed in any asymptomatic carrier or patient with HAM/TSP [56]. The contribution of these highly expanded clones to malignant disease is unknown.

Retroviral gene therapy trials have also shown differential expansion of clonal populations, and in some cases this has led to malignancy. The first two clinical trials of gammaretroviral gene therapy for X-linked severe combined immunodeficiency (SCID-X1) in France and the UK showed effective reconstitution of the patient’s immune system [24,25]. However, within three to five years after treatment, five out of 20 treated patients had developed leukaemia, one of whom subsequently died [3,4]. Seven out of ten patients with Wiskott-Aldrich Syndrome (WAS) treated with gammaretroviral gene therapy also developed acute leukaemia [78,79]. Furthermore, two patients with X-linked chronic granulomatous disorder (X-CGD) in a German trial had successful engraftment but then developed oligoclonal proliferation and myelodysplasia with monosomy 7 [80].

Integration sites near certain growth-promoting genes, including LMO2, were associated with leukaemia in the SCID-X1 trials [3,4,75,81]. Clonal dominance was associated with genes involved in cell cycle control, apoptosis signalling and transcriptional regulation in MLV-based vector integration in mice [82]. These observations raised concerns over the safety of gammaretroviral vectors in gene therapy. However, in contrast to the SCID-X1 trials, out of more than 30 patients with adenosine deaminase deficiency SCID (ADA-SCID) treated with gammaretroviral gene therapy over more than 10 years, none have developed leukaemia [83]. Vector integrations in LMO2 were observed at a frequency similar to that seen in SCID-X1 trials, but LMO2 integration site clones remained <1% of the T cell population during follow up (18 months—six years), implying that integration in these regions alone is not enough to cause malignant transformation [84].

In both gene therapy and exogenous retroviral infections such as HTLV-1, key questions include the following. What is the role of the host genome flanking the integration site in regulating viral or transgene expression? How does this influence cell proliferation or targeting by the host response? Conversely, what is the effect of the introduced viral promoter on the expression of host genes? And finally, what other factors contribute to the skewing of the integration site clone profile?

### 6.2. The Host Immune Response Affects HTLV-1 Integration Site Clone Survival and Abundance

There is little variation in the sequence of HTLV-1, either between clones in an infected person or between different infected individuals [85], compared with other retroviruses such as HIV-1. The sequence conservation reflects the limited infectious spread of HTLV-1, since most detected proviruses have been generated by mitotic division of an infected cell, rather than *de novo* infection and error-prone reverse transcription. Differential expression between integration site clones, therefore, must be predominantly influenced by the host genomic environment of the integration site.

The expression of HTLV-1 viral genes exposes the infected cell to attack by the host immune response. Preferred integration sites *in vitro*, such as regions marked by high levels of epigenetic marks associated with active regulatory elements and sites in close proximity to certain transcription factor binding sites, are less frequent *in vivo* than *in vitro*—although still more frequent than random [39,55]. We have also observed that integration sites *in vivo* are enriched in large genomic regions (1Mb) with high levels of inhibitory marks, compared to *in vitro* integration and *in silico* random sites. It is likely that active integration sites are counter-selected, for instance due to expression of Tax, during the early response to HTLV-1 infection. Studies of acute HTLV-1 infection in humans are rare. However, in a closely related deltaretrovirus, bovine leukaemia virus (BLV), active sites near promoters or genes favoured during initial integration were strongly selected against in the acute phase of the host response [86]. These transcriptionally active sites are likely to favour expression of genes from the integrated BLV provirus [87], making the infected cells a target of the host response.

**Figure 2:**
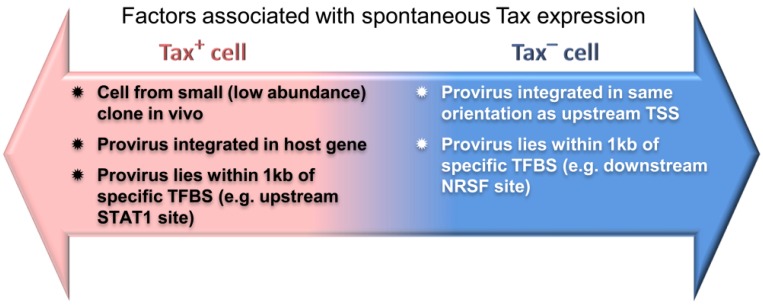
Factors associated with spontaneous HTLV-1 Tax expression in culture. Features of the proviral genomic environment associated with the presence or absence of spontaneous Tax expression by a given clonal population after short-term (18 h) *in vitro* incubation. Reproduced from Figure 7A in [55].

HTLV-1 clones that persist into the chronic phase of HTLV-1 infection proliferate to form clonal populations, while remaining a target of a chronic CTL response. Virtually all infected patients have detectable Tax-specific CTLs [17], and Tax is barely detectable in cells directly *ex vivo* at either the mRNA or protein level. Upon *in vitro* culture the majority (~60%) of cells begin to express Tax within 24 h, although what regulates this release from repression is incompletely understood. However, clones that remained Tax-negative even after overnight *in vitro* culture have a higher clonal abundance *in vivo*. Clones carrying proviruses integrated in the same transcriptional orientation as an upstream flanking gene were more likely to be Tax- silenced *in vitro*, implying that transcriptional interference may silence Tax expression [55]. Tax expression was also associated with integration either upstream or downstream of certain TFBS [55] (Figure 2). Tax silencing appears to be advantageous to the expansion of malignant clones. Approximately 40% of dominant putative malignant clones in ATL have lost Tax expression, through 5’LTR promoter methylation, deletion of the 5’LTR or stop codons within the *tax* gene [56].

Integration sites throughout the acrocentric chromosomes (chr13, 14, 15, 21 and 22) are also significantly over-represented *in vivo* compared to random sites [56]. This does not seem to be an effect of integration targeting, because such sites were not significantly enriched *in vitro*; instead, integration throughout the long arms of the acrocentric chromosomes seems to increase the probability of clone survival *in vivo*. The short arms of the acrocentric chromosomes contain rRNA genes, and take part in formation of the nucleoli. Proximity to the nucleolus (within the perinucleolar region) is associated with silencing, for example the inactive X chromosome [88]: thus, integration in an acrocentric chromosome could provide an advantage to the virus by minimizing exposure to the immune system. Alternatively, expression may be favoured at advantageous times for the virus—for example, following mitosis or in situations of cellular stress.

Although Tax silencing is associated with survival and abundance *in vivo*, clone abundance is also correlated with the frequency of proviruses inserted in genes and actively transcribed areas of the genome during HTLV-1 chronic infection [39,86]. Highly expanded, but presumably non-malignant “intermediate” clones in ATL patients also showed very high frequencies of active epigenetic marks in the surrounding genome, unlike the dominant malignant clone [56]. This observation suggests that transcriptionally active integration sites favour increased proliferation of the infected clone, despite counterselection of highly active sites by the early host response. Since Tax expression is typically silenced *in vivo* and Tax-targeted CTLs are common, it is possible that integration in an active genomic region may instead favour increased expression of HBZ. Integration in transcriptionally active genomic sites were also found to be more frequent in clones from a Southern Japanese asymptomatic cohort compared to clones of a similar abundance in Japanese HAM/TSP patients. This observation may be explained by a requirement for greater clonal proliferation in AC to reach a given absolute abundance, in the face of a stronger host response [58,77].

### 6.3. The Role of Integration Site and Transgene Expression in Gene Therapy Clone Expansion

The transgenes expressed in gene therapy vectors do not have the transactivating effects of the viral proteins, and do not drive unregulated proliferation. Analogous to HTLV-1, however, gene therapy transgene expression may support expansion of transduced cells, and integration site environment may alter transgene expression. Transgene expression may confer a selective advantage on the transduced cell, which is desirable to maintain the transduced population *in vivo* [23]. For instance, the product of the transgene in X-SCID trials, IL2RG, is a subunit for receptors for the cytokines IL-2, 4, 7, 9, 15 and 21, which are required for lymphocyte development and function. Successful expression of the transgene allows proliferation and persistence of transduced cells. Clones of transduced cells expressing higher levels of IL2RG may be positively selected.

The particular transgene expressed may contribute to the risk of adverse events. MLV-based retroviral vectors were used in both SCID-X1 and ADA-SCID trials, yet in contrast to the SCID-X1 trials no recipients in the ADA-SCID trials developed clonal dominance or leukaemia [84,89,90]. This observation raised the possibility that clonal expansion and malignancy are affected by the transgene expressed (IL2RG or ADA respectively). In contrast to IL2RG, the ADA transgene promotes survival rather than growth, by detoxifying the DNA and RNA breakdown products deoxyadenosine and adenosine, which impair development of functional T, B and NK cells. The risk of malignant transformation may be increased in transduced cells carrying a transgene able to stimulate cell proliferation. It is possible that expression of IL2RG co-operates with LMO2 to increase the probability of oncogenic transformation [91]; however, ILR2G was not overexpressed in SCID-X1 leukaemias [75].

In gene therapy, except in diseases involving a severely compromised immune system, such as both SCID-X1 and ADA-SCID, the immune response may also target and restrict transduced cells to expressing a transgene, which encodes a neoantigen, *i.e.*, an antigen “foreign” to the host immune system. Residual retroviral epitopes may provide immunogenic epitopes (“danger” signals) to stimulate the host response [92]. Indeed, humoral immune response have been detected against autologous T cells transduced with chimeric antigen receptors by retroviral transduction [93]. Recent results also indicate that codon optimisation may affect protein conformation and immunogenicity despite retaining primary amino acid sequence (reviewed in [94]). As in HTLV-1, gene therapy clones with elevated transgene expression due to the host genome environment may be preferentially targeted.

At present there is little direct evidence that the characteristics of the integration site of gene therapy vectors alter the expression of the transduced gene. During the Frankfurt CGD trial of two patients, although successful engraftment led to an initial functional recovery, transgene expression became silenced by promoter methylation [80]. Understanding the mechanisms of latency in HIV-1 infections may reveal how the genomic environment affects lentiviral vector silencing *in vivo*. A study comparing HIV-1 latency in five different primary T cell clones or T cell lines found no common predictors of latency across all lines. However, proviruses integrated in the same chromosomal region of the same cell type shared the same pattern of expression (either expressed or silent/inducible), implying an effect of genome location on expression status [95]. The activity of the host cell at the time of integration may influence proviral latency. When HIV-1 integrates in resting cells, proviruses are more frequently found in areas of low gene density compared to integration sites in active cells [60], and viruses in these gene–poor areas appear more likely to be latent. It has also been suggested that the cellular level of NF–kB activity at the time of infection, rather than the site of viral integration, controls establishment of HIV-1 latency in newly infected cells [96].

### 6.4. The Viral Promoter/Enhancer Alters Host Gene Expression and Oligoclonal Proliferation

Insertional mutagenesis by retroviruses, as well as by MLV- and lentiviral-based vectors, has been extensively used to identify oncogenes (reviewed in [97,98]). The promoters or enhancers in the viral vectors de-regulate expression of either oncogenes or tumour suppressor genes, leading to cellular transformation. Gammaretroviral vectors have also been shown to alter nearby host gene expression in gene therapy clinical trials. Overexpression of the proto-oncogene LMO2 was observed in transformed T cells from patients in the French and UK SCID-X1 trials, with integration sites in the proximity of the LMO2 gene [4,75]. LMO2 is a transcription factor that is normally only expressed in hematopoietic stem cells. Transgenic mice expressing Lmo2 in the thymus have shown that Lmo2 is capable of promoting self-renewal of thymocytes, which may facilitate the accumulation of leukemogenic mutations [99]. It seems likely that a major determinant of the malignant expansion seen in these clinical trials was alteration of LMO2 expression by the viral enhancer in the LTR [3,79]. Insertional activation of LMO2 as well as MDS/EV11 was also seen in a patient with WAS who was treated with a gammaretroviral vector [78]. In addition, integration near MDS-EVI1, PRDM16 and SETBP was observed in dominant clones in the gammaretroviral German X-CGD trial. The gammaretroviral vector used in this trial contained a strong enhancer in the SFFV LTR [80,100]. However, in a US trial, which did not use the SFFV LTR, there was no clonal outgrowth despite a transient clinical benefit, suggesting that the enhancer contributed to the malignant outgrowth [101].

While retroviral vector integration in certain sites can clearly be hazardous, there is little evidence of more widespread disturbance of gene expression due to the integrated retroviral gene therapy vectors. Analysis of single T cell clones *ex vivo* from SCID-X1 patients transduced with an IL2GR gammaretroviral vector showed only minor vector-mediated expression changes but no changes in cellular growth rate or mutation rate [102,103]. In T cells isolated from patients treated with gammaretrovirus-based vectors for ADA-SCID, there was a modest increase in the expression of some housekeeping genes near to the vector integration site [84,103]. The LTR might also affect the expression of genes that associate with the integration locus in long-range chromatin interactions. Dysregulation or mutation of the transcription factor c-Myb is associated with T-ALL in humans and with leukaemia in M-MuLV infection of the mouse, following integration either within the gene or at one of several upstream sites. Integration sites upstream of the promoter were found to be long-range enhancers, which interacted with the *c-myb* promoter, demonstrating the ability of integrated retroviruses to influence distant genes [104].

It is interesting that no abnormal clonal expansion has been observed to date in lentiviral vector clinical trials in adrenoleukodystrophy (ALD, 3 patients [28]), Wiskott-Aldrich Syndrome (3 patients [29]), or metachromatic leukodystrophy (3 patients [30]). The more benign behaviour of these vectors may be due to the lentiviral preference for initial integration sites within genes, rather than regulatory elements. Integration within a gene, however, may also disturb physiological expression patterns. Splice variant transcripts from genes around lentiviral vector integration sites have been observed, although this effect was reduced when a self-inactivating version of the vector was used [105,106]. A self-limiting dominant clone in the myeloid compartment was observed in a patient with beta-thalassemia treated with a lentiviral vector. This expansion involved transcriptional activation of the host gene HMGA2 by a vector-truncated transcript generated from a splice variant. However, there was no evidence that this expansion was preleukemic, and it is possible that clonal expansion was limited by the truncated transcript [27]. Recently, two studies have shown that expanded clones from HIV-1-infected patients frequently contain the virus integrated within genes associated with cell growth or mitosis [107,108]. Viral integrations observed within the known hotspots in host genes MKL2 and BACH2, which are associated with clonal expansion, were all present in the same orientation as the respective host gene. Integrations within BACH2 were upstream of the TSS—suggesting an effect on gene expression [107]. Stable integration of HIV-1 can also alter nuclear chromatin organisation: sites of HIV-1 integration tend to occupy a more interior nuclear position than homologous loci [109]. Greater understanding of the mechanisms generating alternative splicing by lentiviral vectors may lead to strategies for recoding lentiviral vector backbones and transgenes, to reduce the probability of altering host gene expression [105,106].

Many lentiviral systems use a self-inactivating vector, in which the promoter within the U3 region of the viral LTR is deleted in both LTRs of the inserted provirus. Transgene expression is driven by an internal promoter [31]. Strong heterologous internal promoters such as SFFV can drive insertional gene activation by self-inactivating lentiviral vectors [110,111]. Human ubiquitously expressed moderate cellular promoters such as PGK and EF1a have a safer profile [110,111], although enhancer activation can be detected in sensitive *in vivo* mouse models [112]. Physiological human promoters can limit unwanted transgene expression, such as the reconstituted WAS gene promoter used in a recent WAS clinical trial [29,110]. Gammaretroviral vectors with a self-inactivating LTR are also being developed [113] and used in an ongoing X-SCID trial with IL2RG expressed from an EF1a promoter [83].

It is clear that insertional mutagenesis contributes to leukaemogenesis caused by gammaretroviral vectors. In contrast, it has been widely believed that the transactivating effects of Tax and HBZ are responsible for leukaemogenesis in HTLV-1 infection [10]. However, a role for insertional mutagenesis in HTLV-1 proliferation and malignant transformation in ATL has not been excluded. This should now be reconsidered in the light of the recent evidence of the very large number of different integration sites present in each infected host. Analogous to MLV and HIV-1, promoters or enhancers within the HTLV–1 LTRs may influence the expression of neighbouring genes. There are no known hotspots of HTLV-1 proviral integration, and cluster analysis has also shown no hotspots of integration significantly associated with leukemic clones [56], although the ontology of the nearest downstream gene was associated with malignant clones in 6% of ATL cases [56]. Although it is plausible that provirally altered neighbouring host gene expression contributes to leukemic clone expansion, it seems likely that oncogenic mutations arise on a background of high proviral load and high virus-driven cell turnover. Dominant (putative malignant) clones in ATL have been observed to arise from the polyclonal background, and not from the existing expanded clones [56,114].

### 6.5. Integration Bias Influences Integration Site Profile in Vivo

It is important to distinguish between sites that are more common because of preferential expansion or survival *in vivo* (whether malignant or benign) and those that are more frequently observed because of intrinsic initial vector targeting preferences. Common integration sites observed in an X-linked ALD lentiviral vector clinical trial and in transduced human hematopoietic stem progenitors cells engrafted into immunodeficient mice both showed broad (megabase) regions where lentiviral integration frequency was enriched. This seems likely to reflect an underlying lentiviral preference for these genomic regions [115]. It is plausible that these regions are active transcriptional regions in hematopoietic stem progenitor cells, as lentiviral vectors show altered integration site preferences depending on the activity of the cell [60,61]. Similarly, gammaretroviral-based vectors have a known preference for integration in active enhancers and promoters, which vary by cell type and development stage [49,116,117]. LMO2 appears to be only a target for MLV integration in CD34^+^ hematopoietic stem cells, which have histone marks reflecting open chromatin at that location [117], and integration near LMO2 is frequently observed even in the absence of *in vivo* selection [49]. The genes associated with oligoclonal proliferation and malignancy in gammaretroviral trials such as LMO2 are frequently described as “proto-oncogenes”—but are also characteristically expressed in stem cells. The higher frequency of integration observed in LMO2 may be in part due to initial integration bias to active regulatory elements, as well as insertional effects [118]. However, there are many complex factors interacting to determine the expansion of a given integration site clone *in vivo*.

### 6.6. Other Causes of Asymmetrical Expansion of Retrovirus-Infected Clones in Vivo

Clones of retrovirus-infected cells can differ in factors other than the integration site that influence survival and proliferation *in vivo*. In T lymphocytes, for example, the specificity of the T cell antigen receptor (TCR) of the infected clone also differs between clones. In chronic co-infection with HTLV-1 and the helminth *Strongyloides stercoralis*, and in patients with bacterial infection in infective dermatitis associated with HTLV-1 (IDH), the most abundant clones are not enriched in transcriptionally active genomic integration sites [57]. It is possible that the expansion of these largest clones is instead driven by TCR recognition of epitopes associated with co-infecting pathogens.

In addition, the conditioning regimen may play an important role in expansion and the risk of adverse events. One difference between the ADA-SCID and SCID-X1 trials was the lack of conditioning in the latter [83]. SCID-X1 trials showed engraftment in the thymus but not bone marrow, in contrast to the ADA-SCID trials. Cell competition in the thymus appears to have a tumour-suppressor role in the thymus, which may have been limited in the absence of T cell progenitor replenishment from the bone marrow in SCID-X1 therapy [119].

Stochastic effects are also likely to play a role in determining which gene therapy integration site clones form the long-term transduced repertoire. For instance, if a heterogeneous population of CD34^+^ cells are transduced, only some will be long-lived hematopoietic stem cells, which will contribute to long-term generation of cells *in vivo*.

## 7. Concluding Thoughts

Long-term clonal integration site composition in both chronic HTLV-1 infection and gene therapy depends on both the initial profile of viral integration and on selective survival and expansion of certain integration sites *in vivo*. In HTLV-1, a key driver of clonal abundance is the expression level of the genes including Tax and HBZ, and the balance between proliferation and immune system targeting. Expansion of gene therapy vector transduced cells can be influenced by insertional mutation, or advantages conferred by certain transgenes, or by the engraftment potential of the cell. In both HTLV-1 infection and in gene therapy, not all clonal expansion is pathogenic, and progression to pathogenesis, such as leukaemia, is the result of a complex integration of factors.

The recent work on HTLV-1 integration has overturned several preconceptions of how the virus behaves *in vitro* and causes disease. It had been widely accepted that oligoclonal proliferation of HTLV–1 infected cells plays a central part in the pathogenesis of both the inflammatory diseases (especially HAM/TSP) and the malignant disease (ATL) caused by HTLV-1. But recent data on clonal abundance and HTLV-1 proviral expression strongly indicate that oligoclonal proliferation *per se* does not contribute to the pathogenesis of either type of disease, even if continuous or intermittent HTLV-1 gene expression is required for viral persistence *in vivo* [56,114]. Additionally, in contrast to previous estimates, there are now known to be thousands of HTLV-1 integration sites in a typical patient. These observations raise the possibility that, although there are no hotspots of integration that favour progression to ATL, insertional mutagenesis may contribute to pathogenesis by providing a polyclonal background on which further oncogenic mutations can arise.

**Figure 3:**
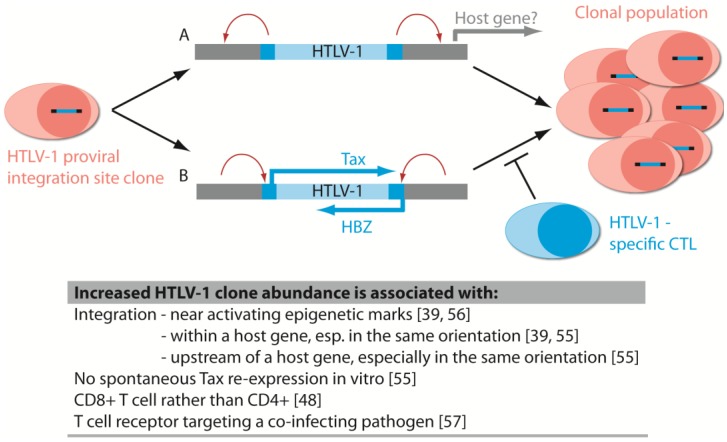
Expansion of HTLV-1 integration site clones *in vivo*. The abundance of HTLV-1 integration site clonal populations during chronic infection may be influenced by (**A**) alteration of host gene expression by viral LTRs and (**B**) features of the genomic environment that alter proviral expression of Tax and HBZ, which can both drive cell proliferation and expose the cell to the cytotoxic lymphocyte (CTL) response. Clone-specific factors not associated with genomic integration site but which may influence clone abundance include the specificity of the T cell antigen receptor.

The focus in the HTLV-1 field to date has been on the role of viral proteins such as Tax and HBZ in driving clonal proliferation and influencing oncogenic progression. The recent observations have revealed the extent of the interaction between host genome and virus in driving viral gene expression, which may be favourable or unfavourable for the virus. The role of the provirus in influencing neighbouring host genes (*i.e.*, insertional mutagenesis) is also gaining attention, analogous to the effects seen with gene therapy vectors and other retroviruses (Figure 3). It is also important to explore potential effects of the provirus on host genes linearly distant on the chromosome but which may be brought into proximity in the nucleus by chromosome folding.

The adverse events in the early gene therapy trials in SCID led to extensive studies to determine the genotoxic safely of retroviral vectors, and the insights from these studies have considerably reduced the side effects of gene therapy. Both benign clonal expansion and malignant transformation in gene therapy are the result of multiple, often interacting, factors, including insertional mutagenesis. The viral integrase determines preferential integration sites; some locations, such as active promoters and enhancers, may increase the chance that integrated sequences alter host gene expression levels. Physiologically appropriate promoters have been used to reduce the risk of vector-mediated host gene expression changes. In addition, the transgene expressed and the conditioning regimen may also support cell survival or proliferation, in turn favouring successful long-term engraftment.

Investigation into the role of the host immune response in limiting clone expansion due to increased expression, as observed in HTLV-1, is becoming more relevant as gene therapy moves into patients with intact immune systems. The host may mount an immune response both to novel or previously unexpressed transgenes that form a neoantigen. More work is also needed on how the host genomic environment of gene therapy vectors influences transgene expression, as in HTLV-1 infection, to optimize the pattern and intensity of transgene expression.

High-throughput integration site analysis allows integration site profiles to be monitored during therapy. LM-PCR was recently used in HIV-1 infection to show that clonal proliferation contributes to the maintenance of the reservoir of HIV-1 during anti-retroviral therapy [107]. In HTLV-1 infection, ATL is notoriously difficult to treat; however, new treatment regimens show promise. Response rates have been improved by a combination of standard chemotherapy regimens with antivirals zidovudine and interferon alpha, although the mechanism of this benefit remains unclear [120], or by treatment with an ADCC-enhanced monoclonal antibody targeting the chemokine receptor CCR4, which is upregulated on ATL cells [121]. On-going work aims to determine what factors contribute to the eventual removal of the dominant clone during therapy, and how that can guide treatment. In gene therapy, analysis of integration sites will further untangle the contribution made by viral vector integration site preference, transgene expressed, and transduced cell type to the risk of adverse effects, and will lead to further improvements in safety and efficacy. Integration site monitoring will also be useful to quantify the diversity and abundance of gene-edited clones in novel gene therapy approaches, such as in transduced keratinocyte skin cells [122].

Our understanding of clonal expansion and malignancy in both HTLV-1 and gene therapy has been instrumental in altering our view of pathogenic mechanisms of HTLV-1 associated diseases, is informing application of novel ATL treatments, and has led to safer gene therapy in the clinic.
